# 

**Published:** 1888-10

**Authors:** 


					﻿THUH^SIX EXTRA PACES IN THIS NUMBER.
Whole Numbei
CCCXXVII.
(Octo bet, 1888.
No. 3**
VOL. XXVIII.
New Seri< s. _
Buffalo
Medical^Surgical
Journal.
Established 1843 by AUSTIN FLINT, N/I. D.
(Jdrtorg :
THOS. LOTHROP, M. D.	W. W. POTTER, M. D.
<&bitars :
7LOYD S. CREGO, M. D. EDWARD B. ANGELL, M. D., Rochester, N.Y.
0
UJ
I
(0
j
CO
1
£
0
z
H
I
r
<
BUFFALO:
i £88.
Entered at the Post-Office at Buffalo, N. Y.. as Second-class Mail Matter.
Times Printing House, Buffalo, N. Y.
				

